# A novel germline (c.314T>A) *SDHB* variant in metastatic paraganglioma: case report and literature review

**DOI:** 10.3389/fendo.2025.1577421

**Published:** 2025-03-27

**Authors:** Stella Bernardi, Stefania Zovato, Gianmaria Pennelli, Marco Cavallaro, Matteo Rovina, Chiara Dobrinja, Alessandra Guglielmi, Fabrizio Zanconati, Daniela Mazzà, Alberto Nieri, Mirco Bartolomei, Francesca Schiavi

**Affiliations:** ^1^ Department of Medical Surgical and Health Sciences, University of Trieste, Trieste, Italy; ^2^ Endocrinology Unit (UCO Medicina Clinica), ASUGI, Cattinara Teaching Hospital, Trieste, Italy; ^3^ Familial Cancer Unit, Veneto Institute of Oncology IOV-IRCCS, Padova, Italy; ^4^ Department of Medicine (DIMED) University of Padua, UOC Anatomia Patologica Azienda Ospedale-Università Padova, Padua, Italy; ^5^ Radiology (UCO Radiologia), ASUGI, Maggiore Hospital, Trieste, Italy; ^6^ Surgery (UCO Clinica Chirurgica), ASUGI, Cattinara Teaching Hospital, Trieste, Italy; ^7^ Oncology (SC Oncologia) ASUGI, Maggiore Hospital, ASUGI, Trieste, Italy; ^8^ Pathology (UCO Anatomia Patologica) ASUGI, Cattinara Teaching Hospital, Trieste, Italy; ^9^ Medical Genetics, Institute for Maternal and Child Health, IRCCS Burlo Garofolo, Trieste, Italy; ^10^ Nuclear Medicine Unit, Onco-Hematological Department, University Hospital of Ferrara, Ferrara, Italy; ^11^ Immunology and Molecular Oncology, Veneto Institute of Oncology IOV-IRCCS, Padova, Italy

**Keywords:** metastatic paraganglioma, SDHB, PPGL, *Averbuch* chemotherapy, PRRT, tyrosine kinase inhibitors, VUS

## Abstract

**Introduction:**

most sympathetic paragangliomas are driven by germline pathogenic variants. Identifying germline succinate dehydrogenase B (*SDHB*) pathogenic variant has important management implications. Here we report a novel germline variant in the *SDHB* gene in a patient with metastatic paraganglioma and his response to available treatments.

**Case presentation:**

a 37-year-old Serbian man was admitted to hospital due to hypertension, tachycardia and hyperhidrosis. Screening for secondary hypertension revealed elevated 24-h urinary normetanephrine. A CT scan showed the presence of a 54 x 76 mm retroperitoneal mass that surrounded the aorta, which was located below the pancreas and behind the duodenum. The patient was diagnosed having extra-adrenal sympathetic metastatic paraganglioma (PGL), for which we scheduled debulking surgery and genetic testing. Tumor debulking improved patient symptoms as well as signs of catecholamine excess and tumor mass effects. Meanwhile waiting for next-generation sequencing (NGS) results, the patient started a treatment with sunitinib. At this point, NGS results showed a novel and previously not reported germline *SDHB* c.314T>A gene variant, which was initially classified as a class 3 variant of uncertain significance. Immunohistochemistry for SDHA and SDHB showed absence of SDHB expression and allowed us to reclassify this variant as a class 4 “likely pathogenic” variant. At this stage, due to disease progression and genetic results, sunitinib was stopped and the patient started peptide receptor radionuclide therapy, which was not able to stop disease progression. In the end, the patient was treated with *Averbuch* chemotherapy (which is still ongoing), with an amelioration of clinical laboratory and imaging parameters.

**Conclusion:**

Clinical characteristics as well as data from SDHB immunohistochemistry well support reclassification of the novel germline *SDHB* c.314T>A gene variant as a class 4 “likely pathogenic” variant in the patient with metastatic PGL. This information might help clinicians in the management of its carriers and their families. In this case, only debulking surgery and chemotherapy with *Averbuch* scheme were clinically effective. Further studies are needed to better clarify and outline at which time point during the disease course *SDHB* patients should start *Averbuch*-scheme chemotherapy.

## 1 Introduction

Pheochromocytomas and paragangliomas (PPGLs) are rare neuroendocrine tumors, affecting 0.6 cases per 100,000 person-years. They derive from the sympathetic or parasympathetic nervous system. In particular, pheochromocytomas arise from the adrenal medulla and paragangliomas arise from either the sympathetic or the parasympathetic paraganglia. Based on their origin, in the first case, they are mainly located in the thorax and the abdomen and they are secreting catecholamines, while in the second case, they are mainly located in the head and the neck and they are generally biochemically silent (1).

From a clinical point of view, PPGLs represent a diagnostic challenge as their presentation varies depending on the clinical effects of elevated catecholamines and/or the multiorgan involvement of tumoral masses (2–4). Diagnosis of PPGLs requires proof of excessive production of catecholamines (now based on the measurement of catecholamine metabolites such as metanephrines and 3-methoxytyramine) as reviewed by Eisenhofer et al. (1), coupled with the anatomical documentation of the tumor by CT or MRI. By contrast, head-and-neck paragangliomas are usually manifested as painless, slowly growing masses, mainly as carotid-body tumors and vagal paragangliomas, or with conductive hearing loss, pulsatile tinnitus and dizziness caused by jugulo-tympanic paragangliomas. Functional imaging is required to search for metastatic disease, as well as for therapeutic purposes.

PPGLs are the tumors with the strongest genetic predisposition known to date (5), given that in almost 80% of patients, PPGLs can be explained by germline or somatic genetic variants. In about 40% of patients, there are germline mutations in one of the 21 known susceptibility genes, while in the remaining 40% of patients there are somatic changes in the same or other genes (5, 6). There is a long list of PPGLs driver genes, the most prevalent being *SDHB SDHD VHL RET* and *NF1* with lower prevalence for *SDHA*, *SDHAF2*, *MAX* and *TMEM127*. Of note, mutations in the *SDHB* gene are associated with the highest risk of metastatic disease among hereditary PPGLs. Other genes involved are *FH*, *MDH2*, *EGLN1* (*PHD2*), *EGLN2* (*PHD1*), *KIF1B*, *IDH3B*, *GOT2*, *SLC25A11*, *DNMT3A*, *DLST*, *EPAS1*, *H3-3A*, *IDH1*, *IDH2*, *CSDE1*, *MAML3*, *FGFR1, HRAS* and *BRAF* (7).

Overall, these genetic mutations promote tumor development through the overexpression of the hypoxia signaling pathways, the activation of kinase receptor signaling pathways, or the activation of the Wnt signaling pathway. Genetic testing is recommended for all patients with PPGLs, as it is crucial for patient management and family screening (8). Next-generation sequencing (NGS) technology has emerged as a valuable tool for this purpose. However, it also generates large amounts of data in need of interpretation, such as in case of genetic variants of unknown significance, which require complementary testing to determine pathogenicity (5). Here, we describe a case of metastatic PGL associated with a novel germline *SDHB* c.314T>A variant, which we reclassified as a “likely pathogenic” variant, and the patient response to treatment.

## 2 Case report

**Figure 1:**
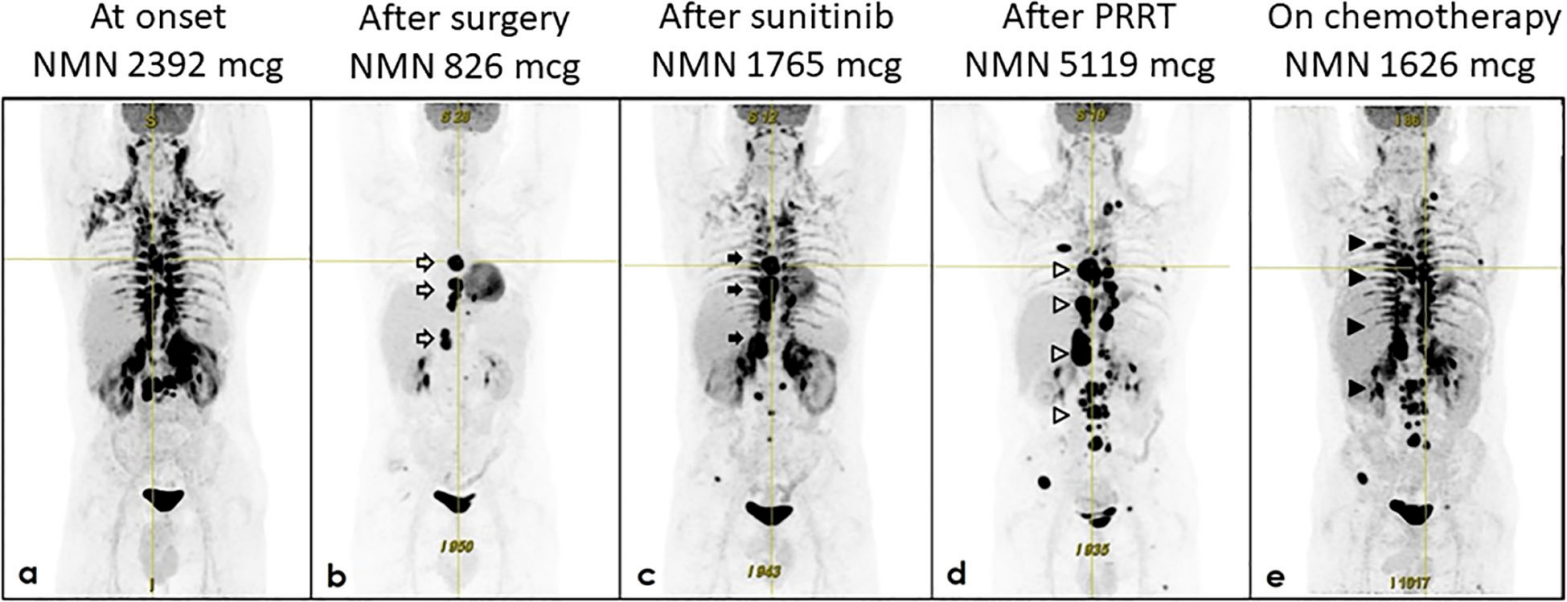
Evolution of biochemical and structural response. The figure shows 24h urinary normetanephrine (NMN) levels and ^18^F-FDG PET/TC scans from onset to date. **(a)** January 2023 at onset; **(b)** February 2023 after surgery; **(c)** October 2023 after sunitinib; **(d)** October 2024 after PRRT; **(e)** January 2025, after 3 cycles of chemotherapy with *Averbuch*-scheme.

In January 2023, a 37-year-old Serbian man was admitted to hospital due to severe hypertension (PA 160/100 mmHg) and tachycardia (120 bpm), associated with hyperhidrosis. He had no other medical conditions or diseases to report. Ambulatory Blood Pressure Monitorng (ABPM) showed severe hypertension with reverse dipping pattern (likely due to postural changes). This was associated with left ventricular hypertrophy, as interventricular septum measured 18 mm, posterior wall thickness measured 14 mm and global longitudinal strain was -15.9% (9). Screening for secondary hypertension revealed that 24-h urinary normetanephrine (NMN) was 2392 mcg (r.v.<43.2), while metanephrine and methoxytyramine were within normal ranges. CgA was 2864nbsp;ng/mL (r.v. < 76.3). A CT-scan showed the presence of a 54 x 76 mm retroperitoneal mass that surrounded the aorta, which was located below the pancreas and behind the duodenum. Several additional thoraco-abdominal masses and metastatic lymph nodes with a maximum diameter of 20 mm were also described. Based on the 2022 WHO update, the patient was diagnosed having metastatic sympathetic abdominal PGL (10). In such cases, ^68^Ga-DOTA-SSA PET/CT should be the first-choice nuclear imaging investigation (11), and, in our patient, it showed tracer uptake in all these lesions. A parallel ^18^F-FDG PET/CT showed tracer uptake in the same lesions (**Figure 1a**).

Given the expression of somatostatin receptors and a few case reports indicating the use of long-acting cold somatostatin analog in similar cases (12–14), treatment with octreotide was initiated in addition to α-adrenoceptor blockade and other antihypertensive drugs. Then, in February 2023, the patient underwent debulking surgery, i.e. enbloc resection of the para-aortic mass with the para-aortic lymph nodes (15). Histologic examination was consistent with the diagnosis of metastatic PGL, as it showed positive expression of chromogranin and synaptophysin and the presence of ribbons of epithelioid chief cells divided by fibrous bands in the para-aortic mass as well as in two lymph nodes. Ki-67 proliferation index was 15%. Debulking surgery led to a reduction of 24-h urinary NMN to 826.8 mcg. This was associated with blood pressure improvement and the patient was discharged with doxazosin 12 mg/day, bisoprolol 2.5 mg/day, amlodipine 5 mg/day, and ramipril 5 mg/day. Post-operative ^18^F-FDG PET/CT showed tracer uptake in other paraganglia (**Figure 1b**).

PPGLs are the tumor with the highest reported degree of heritability, and guidelines recommend considering genetic counselling and testing for any patient diagnosed with it (5, 8). Consistent with this recommendation, the patient underwent genetic testing despite a negative family history for PPGL or other manifestations associated with syndromes in which PPGL is a known feature. Then, in March 2023, while waiting for NGS results, we started the treatment with the tyrosine kinase inhibitor (TKI) sunitinib, as metastatic PPGLs can benefit from TKI therapy (16). Sunitinib is usually recommended as a treatment option for slowly or moderately progressing *SDHB* PPGLs (17). However, at that time we did not know yet the gentic profile of the disease nor its rate of progression. Sunitinib was started at a dose of 25 mg/day and then was increased to 37.5 mg/day. This was associated with blood pressure worsening and it required an increase of the antihypertensive therapy to doxazosin 20 mg/day, carvedilol 12.5 mg/day, amlodipine 10 mg/day, and olmesartan 40 mg/day. A few months later, 24-h urinary NMN was 1765 mcg, and a new CT-scan showed an enlargement of a thoracic lesion from 25 x 35 x 36 mm (March 2023) to 30 x 37 x 40 mm (August 2023) as well as the enlargement of a retrocrural mass measuring 26 x 24 x 27 mm. The ^18^F-FDG PET/CT confirmed disease progression (**Figure 1c**).

**Figure 2:**
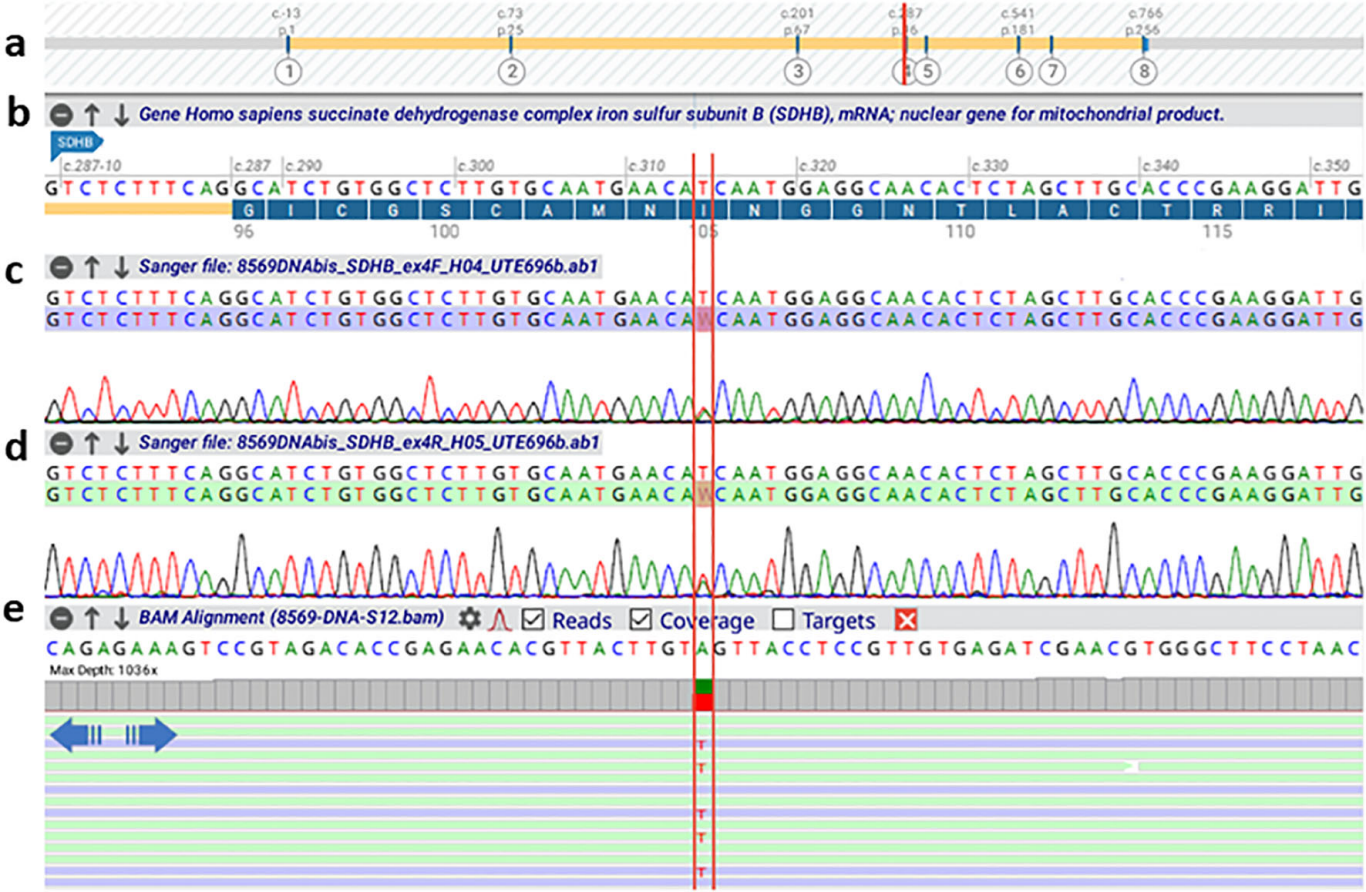
Alamut Visual Plus alignment of the BAM file (NGS sequencing) and AB1 files (Sanger sequencing) for the region harboring the SDHB c.314T>A variation. The image provides a comparative analysis of the two sequencing technologies, with the nucleotide substitution site in the SDHB gene highlighted in red. **(a)** Schematic representation of the SDHB gene structure. **(b)** Zoomed-in view of exon 4, showing the region harboring the variation. **(c, d)** Electropherograms from Sanger sequencing of the forward and reverse strands, respectively. **(e)** NGS sequencing data, including coverage and alignment tracks.

At this point, NGS results identified a novel, previously unreported germline variant in the *succinate dehydrogenase subunit B* (*SDHB)* gene. Specifically, we detected the variant NM_003000.3(*SDHB*):c.314T>A p.(Ile105Asn) in heterozygous state (**Figure 2**). In particular, with respect to the panel of genes that was analyzed with NGS, the coding exons and flanking intronic regions (at least 25 bp) of 81 genes associated with endocrine, renal, and gastrointestinal hereditary cancers were enriched using a hybrid-capture approach (Twist Library Prep EF Kit 2.0 and Custom Hybridization Capture Panels “TE-ERGI v2” - Twist Bioscience). Library preparation was followed by paired-end sequencing (2 × 251 cycles) on the Illumina MiSeq system using v3 chemistry. A secondary analysis was performed using Sophia DDM software (Sophia Genetics SA) to detect single nucleotide variants (SNVs), insertions/deletions (indels), and copy number variations (CNVs). The genes included in the virtual PPGL panel were: VHL, BRK1 (limited to CNV analysis), RET, NF1, SDHA, SDHAF2, SDHB, SDHC, SDHD, TMEM127, MAX, FH, DNMT3A, EGLN1, EGLN2, EPAS1, MDH2, MEN1, SLC25A11, DLST, REXO2, MYO5B, GOT2, and IDH3B. The NGS test was developed and validated in-house as a Laboratory Developed Test, demonstrating an analytical sensitivity and specificity of >99%. The identified variant c.314T>A in SDHB gene was confirmed in an independent DNA sample using Sanger sequencing.

**Figure 3:**
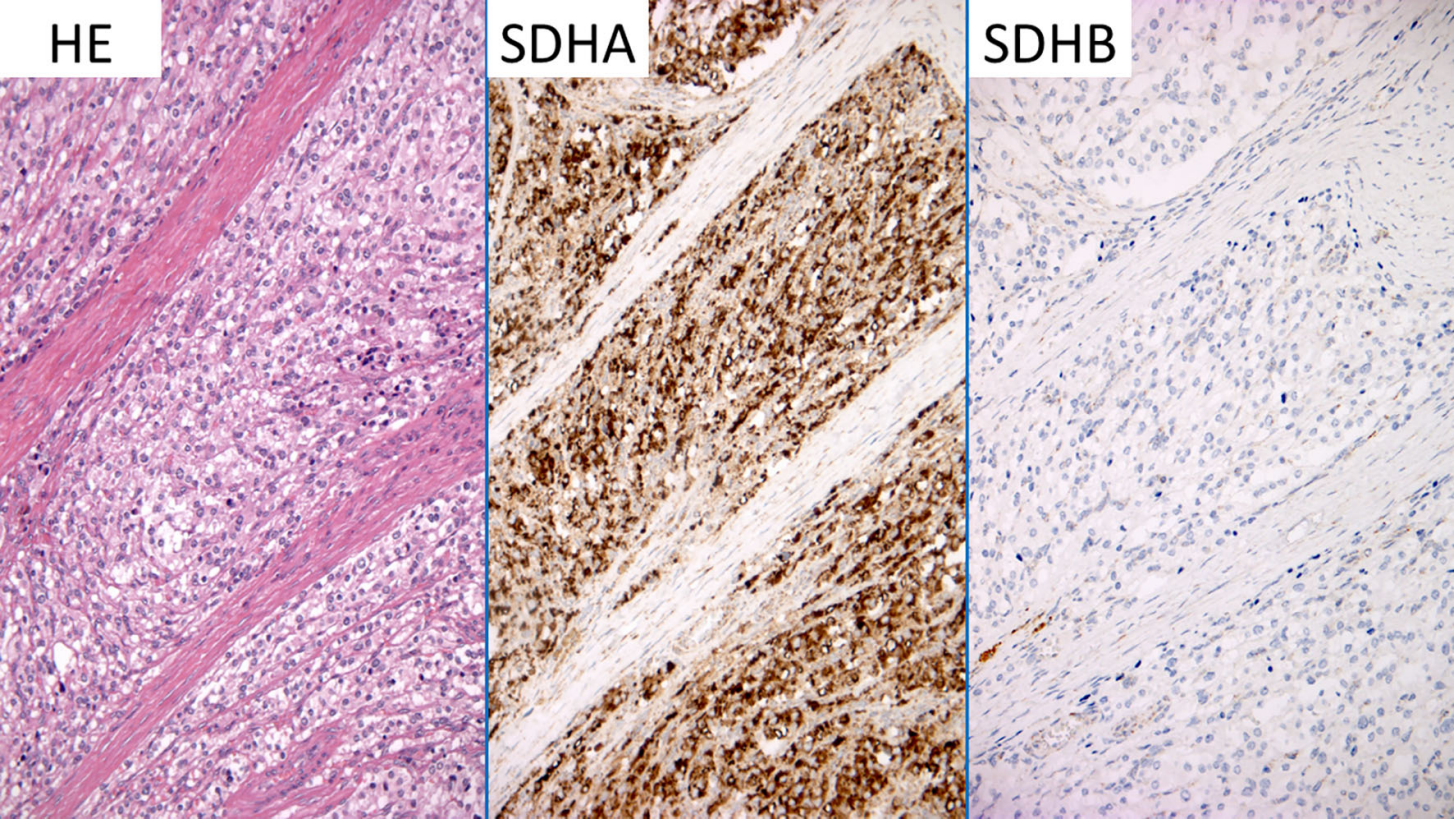
SDHA SDHB immunostaining. The figure shows different stainings performed on slides of the tumoral mass, which was surgically removed, to order to clarify the significance of (c.314T>A) *SDHB* gene variant. HE, hematoxylin and eosin; SDHA, succinate dehydrogenase A immunostaining; SDHB, succinate dehydrogenase B immunostaining.

Based on the recommendations of the American College of Medical Genetics and Genomics (18), and on the CanVIG-UK SDHB/D Gene-Specific Guidance v.1.3 (19), this variant was initially classified as a variant of uncertain significance for insufficient evidence toward pathogenicity (class 3), as it was absent in controls (GnomAD v.4.0) and in locus specific databases (LOVD SDHB and ClinVar). Nevertheless, this nucleotide change had a REVEL score of 0.92, which exceeds the 0.7 threshold (20), supporting a deleterious effect on the gene product based on *in silico* predictions. In order to collect additional evidence toward pathogenicity, immunohistochemistry for SDHA and SDHB was performed and it showed absence of SDHB (**Figure 3**). Based on these data, following the specific recommendations for *SDHB* gene variant interpretation (18, 19), the variant *SDHB* c.314T>A, p.(Ile105Asn) was re-classified as a class 4 “likely pathogenic” variant. At this point, we screened the patient siblings (a brother) and his children with an age > 5 years (two kids), who were found negative.

**Figure 4:**
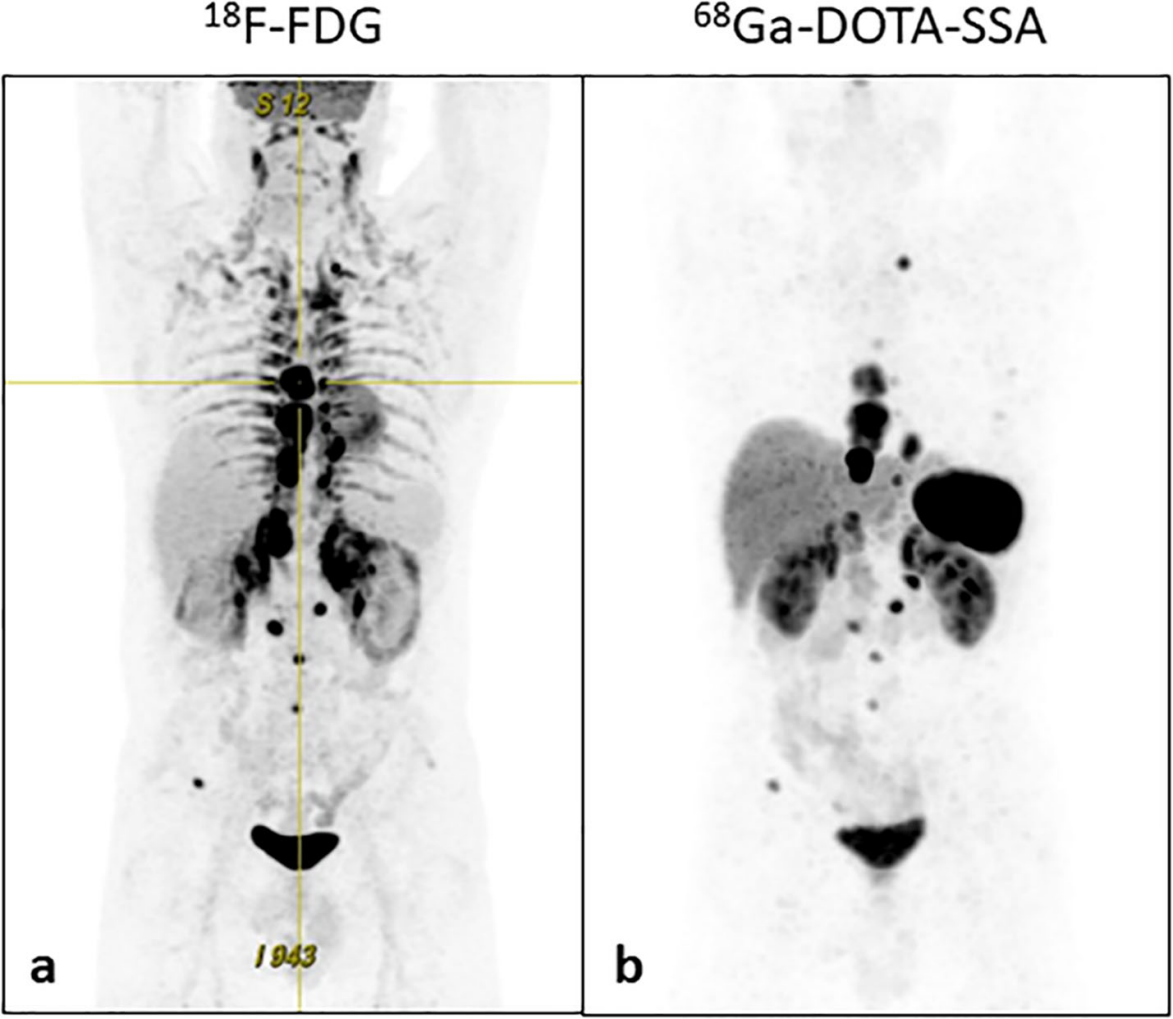
18F-FDG and 68Ga-DOTA-SSA PET/CT images before PRRT. The figure shows **(a)** ^18^F-FDG PCT/TC scan on the left and **(b)** ^68^Ga-DOTA-SSA PET/CT scan on the right.

Taking into account not only NGS results but also the disease progression, sunitinib and octreotide were stopped and, in December 2023, the patient started peptide receptor radionuclide therapy (PRRT) with Yttrium-90 [^90^Y]-DOTATOC and Lutetium-177 [^177^Lu]-DOTATOC. **Figure 4** shows ^18^F-FDG and ^68^Ga-DOTA-SSA PET/CT scans obtained before PRRT. The patients underwent 5 cycles from December 2023 to July 2024. Total cumulative activity was 5.49 GBq from [^90^Y]-DOTATOC and 16.04 GBq from [^177^Lu]-DOTATOC. This therapy was well tolerated. However, although initially there was an improvement in tumor related symptoms, in October 2024 the patient had a symptom relapse that was associated with biochemical and structural disease progression (**Figure 1d**). Antihypertensive therapy was changed to doxazosin 16 mg/day, bisoprolol 15 mg/day, amlodipine 10 mg/day, and olmesartan 40 mg/day. 24-h urinary NMN was 5119 mcg.

For this reason, in November 2024, the patient started chemotherapy with the *Averbuch*-scheme with a rapid amelioration of symptoms, as well as biochemical and structural partial response. This is consistent with another recent case report (21). Finally, in January 2025, after 3 cycles, 24-h urinary NMN was 1626 mcg. ^18^F-FDG-PET showed partial response (**Figure 1e**), i.e. reduction of the number, size and tracer uptake in the paraganglia and bone, and reduction of tracer uptake in the lung. The chemotherapy is still ongoing.

## 3 Discussion

Most PPGLs are driven by germline pathogenic variants. Mutations in the *SDH*x genes (*SDHA*, *SDHB*, *SDHC*, and *SDHD*), which encode the four subunits of the mitochondrial enzyme succinate dehydrogenase (SDH), are associated with a predisposition for developing hereditary PPGLs. The *SDHB* pathogenic variants have an estimated disease penetrance of 20-30% by the age of 65 years (22). Nevertheless, *SDHB* PPGLs seem to have the highest rates of disease-specific morbidity and mortality compared with other hereditary PPGLs (17). The patients with a PPGL who have a *SDHB* mutation, large tumor size (>5 cm), extra-adrenal location, dopaminergic phenotype, and high Ki-67 have a higher risk of developing metastases (15). In a recent retrospective study the median overall survival of patients with metastatic PPGLs was 6.7 years and the 5-year overall survival was 62% (23). Interestingly, in the study by Hescot et al., hypersecretion, rather than *SDHB* mutations, was identified as an independent significant prognostic factor of worst overall survival. Metastatic PPGLs with a *SDHB* mutation require a timely multidisciplinary approach to ensure high-quality care (17). A recent consensus statement has addressed the issue of the management of *SDHB* PPGLs, recommending an individualized approach based on patient general conditions, disease growth rate, tumor burden, and patient symptoms. Treatments include (debulking) surgery, local therapies, PRRT or [^131^I]-MIBG therapy, TKI and chemotherapy (17).

Here, we describe a novel “likely pathogenic” *SDHB* germline variant, c.314T>A, p.(Ile105Asn), in a patient with metastatic PGL. All patients with PPGLs should undergo genetic counselling and testing, as identifying a genetic predisposition is key to patient management (8), first of all because this is an indication to lifelong follow-up. Consistent with these recommendations, genetic testing was found having a positive impact on PPGL management and outcomes (24). In particular, Buffet et al. showed that patients who were informed of their genetic status within the year following the first PPGL diagnosis received better follow-up and had better 5-year survival rate after the discovery of metachronous metastases, as compared to patients who received the genetic test several years after initial PPGL diagnosis (24). Secondly, given that these mutations are inherited in an autosomal dominant fashion, the identification of a germline mutation should prompt genetic testing in first-degree family members (8).

NGS is the preferred technique to analyze all relevant genes in a single test (5). Nevertheless, this technique generates large amounts of data in need of interpretation, such as genetic variants of unknown/uncertain significance (VUS) that require complementary testing to establish whether a mutation is pathogenic (5). Classification of VUS to a category with a defined clinical significance is key to the management of the patient and their first-degree family members, as full surveillance applies only to carriers of a pathogenic mutation. Today, there are several potential approaches to assess functionality and establish the pathogenic role of any VUS (25). They include SDHB immunohistochemistry (26), whose sensitivity and specificity are 100% and 84% (26), and mass-spectrometry-based metabolomics of Krebs cycle intermediates (27), which seems to have a higher specificity than SDHB immunohistochemistry (28). In our case, we relied on SDHB immunohistochemistry only to confirm the functional impact of the variant.

It is current opinion that the relevance of the genetic analysis goes beyond the indication to lifelong follow-up, or screening of family members, as it may extend to patient therapy, facilitating a cluster-specific (personalized) patient management plan (15). In particular, based on the type of gene mutations, PPGLs have been recently classified into 3 specific molecular clusters (15): pseudohypoxia-related clusters 1A and 1B; kinase signaling-related cluster 2; and Wnt signaling-related cluster 3. Among them, cluster 1A-related PPGLs include *SDHB* mutations as well as other mutations in Krebs cycle-associated genes, such as *SDHx*, *FH*, *MDH2*, *IDH*, *GOT2*, *SLC25A11* and *DLST*. Cluster 1A-related PPGLs tend to have a more aggressive phenotype, and they have a metastatic risk of 40%, with the highest risk in case of *SDHB* mutations (35-75%). They tend to have a noradrenergic phenotype and they are more likely to be associated with lower basic symptom scores and sustained hypertension. In addition, due to higher expression of somatostatin receptors the most sensitive imaging method is [^68^Ga]-DOTA-SSA PET/CT with a sensitivity of 94% to 100% (11).

Interestingly, in the cluster 1-related PPGLs some therapeutics seem to be more effective than others (15). These include PRRT in case of PPGLs with slow to moderate progression, or chemotherapy (*Averbuch*-scheme, temozolomide with or without poly(ADP-ribose)polymerase inhibitors) in case of rapid progression. By contrast, (HSA)/conventional [^131^I]-MIBG therapy or TKI (sunitinib and cabozantinib) seem to be more effective in cluster 2-related PPGLs. It is also recommended that in patients with more rapidly progressing metastatic PPGL including those with a higher Ki-67, *Averbuch*-scheme chemotherapy rather than TKI or PRRT are used as the first line of treatment (15). Consistent with this concept, our patient did not respond well to sunitinib nor PRRT initially. He did not respond to octreotide either, but with respect to this drug, which is still commonly administered in PPGLs (21), its use is not supported by current guidelines (14, 17, 29).

PRRT agents consist of a radionuclide, such as 177-Lutetium (^177^Lu) or 90-Yttrium (^90^Y) that is linked to a peptide acting as a somatostatin receptor agonist, such as TOC-Tyr3 Octreotide or TATE-Tyr3 Octreotide, with the help of a chelator (DOTA). The agents bind to the somatostatin receptors on the tumor cell membranes, delivering radiation from the β-emitting radionuclides and causing cellular damage (30). Since our first report on the efficacy of PRRT in 4 patients with hereditary nonmetastatic PGLs (31), different retrospective studies have confirmed PRRT favorable effect in advanced or metastatic PPGLs. In addition, very recently, Rubino et al. (32) have demonstrated that in 30 patients with locally advanced or metastatic PPGLs, PRRT led to partial response in 23% of patients, stable disease in 63%, and an overall disease control rate of 86%. In line with these findings, two meta-analyses have shown that PRRT is a safe and efficacious treatment option for advanced PPGLs (30, 33). Nevertheless, in these works mutational status was either not specified (30) or *SDHB* gene variants were present only in a minority of patients, like in the study by Marretta, where it was present in 43 out of 201 patients (21%) (33). In a recent consensus paper on molecular imaging and theranostics in neuroendocrine neoplasms, consensus was not reached on the necessity of using genetic examination to choose the appropriate radiopharmaceutical for patients with inoperable or metastatic PPGLs (34). However, patients with *SDHB* mutations seem to have a worse overall survival and progression free survival than other groups after PRRT (35), which is consistent with the absence of response to PRRT of the *SDHB* patient described in this report. Thus, additional well-conducted clinical studies or well-designed clinical trials are needed to better clarify the response to PRRT based on mutational status of patients with progressive metastatic PPGLs including those with *SDHx* mutations.

Later on, during the disease course, the patient was treated with the *Averbuch*-scheme (cyclophosphamide, vincristine, dacarbazine, CVD) chemotherapy, which was originally chosen to treat PPGLs because its components are effective in neuroblastoma (36). A recent international expert consensus statement suggests this type of treatment in patients with PPGLs and *SDHB* pathogenic variants when tumor growth rate is fast, tumor burden is high, and patients are symptomatic (17). A systematic review and meta-analysis has shown that this type of chemotherapy led to a partial response concerning tumor volume in about 37% of patients and to a partial response on catecholamine excess in about 40% of patients with PPGLs (37). Nevertheless, response may vary based on genetic mutations. For instance, more recently, Jawed et al. documented that in 12 patients with only *SDHB* PPGLs, CVD was always associated with tumor reduction (12-100% by RECIST) (38). In their study, complete response was seen in two patients, while partial response was observed in 8 patients (38). In addition, Fishbein et al. showed that *SDHB* mutation carriers responded better to CVD than non-mutation carriers. The Authors concluded that if these results are confirmed in larger prospective cohorts, this could affect choice and possibly timing of chemotherapy in *SDHB* patients with consideration to giving CVD earlier in their treatment plan (39). This reinforces the notion that assessing the mutational status of PPGL patients is key for their management.

In conclusion, here we describe the case of a patient with metastatic sympathetic abdominal PGL carrying the novel germline *SDHB* c.314T>A p.(Ile105Asn) gene variant, classified likely pathogenic based on patient’s phenotype and data from SDHB immunohistochemistry (class 4). This information might assist clinicians in the management of its carriers and their families. Moreover, in this case, only debulking surgery and chemotherapy with *Averbuch*-scheme were effective. Case reports can provide real-world evidence on rare diseases, however, they cannot deliver quantitative data nor can they allow generalizations (40). In addition, limitations of our case include not having metabolite profiling of tumor tissue, genetic screening of the whole patient’s family, and the fact that chemotherapy is still ongoing. Further studies are needed to better clarify and outline at which time point during the disease course *SDHB* patients should start *Averbuch*-scheme chemotherapy.

## Data Availability

The raw data supporting the conclusions of this article will be made available by the authors, without undue reservation.
